# Exploration of the Regulatory Mechanism of Secondary Metabolism by Comparative Transcriptomics in *Aspergillus flavus*

**DOI:** 10.3389/fmicb.2018.01568

**Published:** 2018-08-07

**Authors:** Guangshan Yao, Yuewei Yue, Yishi Fu, Zhou Fang, Zhangling Xu, Genli Ma, Shihua Wang

**Affiliations:** Fujian Key Laboratory of Pathogenic Fungi and Mycotoxins, Key Laboratory of Biopesticide and Chemical Biology of Ministry of Education, School of Life Sciences, Fujian Agriculture and Forestry University, Fuzhou, China

**Keywords:** *Aspergillus flavus*, aflatoxin, transcriptome, LaeA-like methyltransferase, RNA-seq

## Abstract

Mycotoxins cause a huge threaten to agriculture, food safety, and human and animal life. Among them, aflatoxins (AFs) have always been considered the most potent carcinogens, and filamentous fungi from *Aspergillus* genus are their major producers, especially *A. flavus*. Although the biosynthesis path of these chemicals had been well-identified, the regulatory mechanisms controlling expression of AF gene cluster were poorly understood. In this report, genome-wide transcriptome profiles of *A. flavus* from AF conducing [yeast sucrose media (YES)] and non-conducing [yeast peptone media (YEP)] conditions were compared by using deep RNA sequencing (RNA-seq), and the results revealed that AF biosynthesis pathway and biosynthesis of amino acids were significantly upregulated in YES vs. YEP. Further, a novel LaeA-like methyltransferase AFLA_121330 (Lael1) was identified for the first time, to play a specific role in the regulation of AF biosynthesis. Contrary to LaeA, which gene deletion reduced the level, *lael1* deletion resulted in a significant increase in AF production. Further, co-expression network analysis revealed that mitochondrial pyruvate transport and signal peptide processing were potentially involved in AF synthesis for the first time, as well as biological processes of ribosome, branched-chain amino acid biosynthetic process and translation were co-regulated by AfRafA and AfStuA. To sum up, our analyses could provide novel insights into the molecular mechanism for controlling the AF and other secondary metabolite synthesis, adding novel targets for plant breeding and making fungicides.

## Introduction

Aflatoxins (AFs) has always been labeled as the most potent carcinogens (Squire, 1981; Amaike and Keller, 2011). In general, AF and its major producer *Aspergillus flavus* have been frequently detected in oil-enriched seeds such as peanuts, maize seeds, and walnuts (Bhatnagar-Mathur et al., 2015). However, a recent fear was that aflatoxin B1 (AFB1) or aflatoxin M1(AFM1) were widely detected in our daily food on a global scale, including their presence in rice and rice products in Pakistan (Iqbal et al., 2016), sugarcane juice in Egypt (Abdallah et al., 2016), sausages in Croatia (Kabak and Dobson, 2017), milk in Europe (Kumar et al., 2017b), vegetable oil (Sun et al., 2011) and Chinese traditional medicines in China (Zhao et al., 2016), and even human breast milk in Turkey (Kiliç Altun et al., 2017). An increasing body of evidences demonstrated that exposure to or ingestion of AF severely impaired human or animal health (Kumar et al., 2017b). Long time ingestion of AF-containing food or food adducts are associated with high rates in hepatocellular carcinoma (Pitt and Miller, 2017). Even worse, several lines of evidences suggested that AF uptake and hepatitis B virus synergistically induced the development of liver cancer (Kew, 2003; Aydin et al., 2015; Chu et al., 2017). Therefore, an improved understanding of AF synthesis and metabolism, and where its regulatory mechanism is urgently required, which would greatly contribute to the development of new and effective long-term management strategies to avoid the severe effects caused by these toxic chemicals.

Until now, the AF biosynthetic pathway had been essentially clarified (Roze et al., 2013), however, the regulatory mechanism that orchestrating the AF cluster gene expression remains largely unknown. Nutrient significantly impact the synthesis of AF, and yeast sucrose media (YES) containing high concentration sucrose could induce the formation of AFs, but yeast peptone media (YEP) could not (Fountain et al., 2015). Paradoxically, an earlier report found that addition of peptone in a chemical defined media increased AFs (Reddy et al., 1979). Compared to other nitrogen source, glutamine was reported to be the best one to induce the toxin production (Wang et al., 2017). As the secondary metabolism from *Aspergillus*, biosynthesis of AFs was affected and governed by various environmental cues and a number of proteins at multiple levels, including chromosome, transcription, and post-translation modifications *in vivo* (Amaike and Keller, 2011; Amare and Keller, 2014; Calvo and Cary, 2015). A screen of sterigmatocystin (the precursor of AFs) mutants in *A. nidulans* lead to the identification of an essential regulator LaeA (Bok and Keller, 2004). It is well-known that LaeA, containing a methyltransferase domain, functions as a global regulator of secondary metabolism in various filamentous fungi (Bok and Keller, 2004; Kale et al., 2008; Kosalkova et al., 2009; Chettri and Bradshaw, 2016; Kumar et al., 2017a), especially toxicogenic *A. flavus* (Kale et al., 2008). And, a systematic transcriptome analysis revealed that LaeA affected expression of 26 of 55 secondary metabolite biosynthetic clusters in *A. flavus* (Georgianna et al., 2010). Recently, LlmF, a laeA-like protein, was reported to negatively regulate the production of sterigmatocystin production in *A. nidulans* by modulating the nuclear import of VeA in *A. nidulans* (Palmer et al., 2013). As in *A. nidulans*, a number of novel proteins containing a methyltransferases domain showed high similarity to LaeA in *A. flavus*. Until now, only the role of LaeA in AF synthesis had been confirmed (Bok and Keller, 2004; Kale et al., 2008), but other LaeA-like methyltransferases remains unidentified in *A. flavus*.

Pathway-specific and global transcription factors (TFs) were also reported to critically involve in the regulation of AF gene cluster expression. AflR, as the AF pathway-specific TF, is absolutely required for expression of most of the genes in the AF cluster (Ehrlich et al., 1999). And, AflS (previously named as AflJ) was reported to interact with, and activate AflR to exert its regulatory role (Chang, 2003). It was now well known that fungal development was closely associated with biosynthesis of secondary metabolism (Amare and Keller, 2014; Calvo and Cary, 2015). It was recently found that several TFs co-regulated the conidiation, sclerotial development and AF formation in *A. flavus*, including MtfA (Zhuang et al., 2016), RtfA (Lohmar et al., 2016), and NsdC (Gilbert et al., 2016). More recently, we had identified two APSES TFs AfRafA and AfStuA to be required for fungal conidial and sclerotial development, and colonization of plants. Further, loss of AfStuA completely inhibited the biosynthesis of mycotoxins including AFs and cyclopiazonic acid, and Δ*AfRafA* reduced AF, but enhanced the cyclopiazonic acid biosynthesis (Yao et al., 2017). Therefore, it is reasonable to speculate that AfRafA and AfStuA mediate a complex regulatory network with crucial roles in multiple cellular processes in *A. flavus*. Undoubtedly, comprehensively identifying their downstream genes or pathways contributes to obtain novel regulators for toxin biosynthesis or fungal pathogenicity.

In the present report, we comprehensively compared the transcriptomes of *A. flavus* WT strains culturing in AF inducing vs. non-inducing conditions, Δ*AfRafA* vs. WT, and Δ*AfStuA* vs. WT. Our results revealed that amino acid biosynthesis and metabolism were significantly activated under YES relative to YEP and subjected to regulation of both AfRafA and AfStuA. Weighted correlation network analysis of differentially expressed genes reveal that AfRafA and AfStuA coordinately control novel cellular processes with potential roles in AF biosynthesis. Further, many AF regulators were found to be regulated by AfRafA or AfStuA, or both, and activated in YES vs. YEP. In addition, our comparative transcriptome suggested that expression of many SM gene clusters differentially response to AfRafA and AfStuA. Most importantly, a novel LaeA-like protein Lael1 was demonstrated to have a specific role in regulation of the AF expression.

## Materials and Methods

### Strains and Culture Conditions

The strains used in this study including WT, Δ*AfRafA*, and Δ*AfStuA* were stored in our lab constructed previously (Yao et al., 2017). The strain PTSΔku70ΔpyrG (Chang et al., 2010) was used as the recipient strain to generate the deletion mutants for gene *AFLA_121330*. All *A. flavus* strains were cultured onto Potato dextrose agar (PDA, BD Difco, United States) to obtain mycelia and conidia, and then stored in 30% glycerol solution at -70°C. When compared AF synthesis, YES (2% yeast extract, 15% sucrose, and 0.1% MgSO_4_) and GMM (Glucose 10 g/L, NaNO_3_ 6 g, KCl 0.52 g/L, MgSO_4_.7H_2_O 0.52 g/L, KH_2_PO_4_ 1.52 g/L, and added 1 mL trace elements solution per liter) with 5 mM glutamine (Shimizu and Keller, 2001), and YEP (2% yeast extract, 15% peptone) represents the AF conducing and non-inducing conditions, respectively.

### Determination of AF Production via TLC and HPLC

Extraction and determination of AFs were performed as previously described (Yao et al., 2017). The AFs were extracted by using chloroform. TLC analysis of AFB1 was performed with the acetone:chloroform (1:9, v/v) solvent system, and AFB1 spots were displayed under ultraviolet activation at 365 nm. HPLC analysis of AFB1 was conducted by using the Waters HPLC 1525 system (Waters, United States) equipped with a MYCOTOX^TM^ reversed-phase C18 column (5 μm, 4.6 mm × 150 mm) and a fluorescent detector (λ = 365 nm, λ = 430 nm). Firstly, the column was equilibrated in the mobile phase (water:methanol:acetonitrile, 56:22:22) at 42°C for 1 h. Each chloroform extract was re-dissolved in methanol, filtered through a 0.22 μm nylon filter membrane, and then separated in a 100% mobile phase at a flow rate of 1.0 mL/min. The AFB1 concentration of each sample was counted by using a calibration curves and the AFB1 standard (HPLC grade) were purchased from Sigma (Sigma, Germany).

### Gene Deletion and Complementation

Extraction of genomic DNA of *A. flavus* and standard PCR were performed as previously described (Yu et al., 2004). The AfRafA and AfStuA deletion strains used were constructed previously (Yao et al., 2017) and the *Lael1* deletion strains was generated in the present study. Double-joint PCR was used to construct a gene deletion cassette, using the *pyrG* gene amplified from *A. fumigatus* as the selectable marker. All primers that were used to amplify the 5′- and 3′-flanks were listed in **Supplementary Table S2** with the *A. flavus* gDNA as template. The entire gene deletion cassette was amplified with specific primers, using the 5′- and 3′-flanks for gene *AFLA_121330*, and *pyrG* mix as template. The PCR products were transformed into the protoplasts of PTSΔ*ku70*Δ*pyrG*. Protoplast preparation and PEG-mediated fungal transformation were performed following previously described methods (Chang, 2008). Diagnostic PCR and RT-PCR were used to identify the positive transformants. To construct the complementation strain, an expression cassette containing promoter, coding region, and terminator was amplified by using high-fidelity pfu DNA polymerase (TransGen, Beijing, China). Purified expression cassette together with plasmid pTRI with pyrithiamine (*ptrA*) as the selectable marker (Chang et al., 2010), were co-transformed into the protoplast of the deleted strain. All primers used are listed in **Table 1**.

**Table 1:** Primers used in this study.

Primer name	Sequence 5′–3′
AFLA_121330F1	AATGGAGCACCCACTTTGAC
AFLA_121330F2	ATGGACGATAAATCAAACGA
AFLA_121330R1	ATCGCAGATAGTTTAGCACC
AFLA_121330R2	AGAATACGAGGCGAACAATA
AFLA121330pyrGR	GGGTGAAGAGCATTGTTTGAG GCTCTTGTTCAAGAATTGCGGA
AFLA121330pyrGF	GCATCAGTGCCTCCTCTCAGA CCTGCCTACCGATGATCGATA
PyrGF	GCCTCAAACAATGCTCTTCACCC
PyrGR	GTCTGAGAGGAGGCACTGATGC
AFLA_121330RTF	TCAACTGCTTCTCCGACGAT
AFLA_121331RTR	CAATTCCTTCCGCATACCTG

### RNA Extraction

*Aspergillus flavus* strains were inoculated into YES liquid media and pre-cultured for 24 h, and then transferred into fresh YES media or YEP for stationary culture at 29°C for 48 h. The collected mycelia were frozen in liquid nitrogen, and stored under -80°C conditions. RNA extraction was performed using an RNA reagent Kit (TRIzol reagent, Biomarker Technologies, China) and following the protocols of the manufacture. The quality and integrity of RNA samples were determined using Nanodrop and Agilent 2100 bioanalyzer (Agilent Technologies, Palo Alto, CA, United States), respectively, while the quantity was determined with a Qubit RNA assay kit (Life Invitrogen, United States).

### RNA-seq and Enrichment Analysis of Differentially Expressed Genes

The total RNA of three biological replicates for Δ*AfRafA*, Δ*AfStuA*, and WT grown in YES and YEP was sequenced. Libraries were prepared according to standard protocols from Illumina Inc. (San Diego, CA, United States) and sequenced on a HiSeq 2000 platform (Novogene, Beijing, China). Low-quality reads (Phred ≤ 20) and adaptor sequences were filtered out, and the Q20, Q30, and GC content of the clean data were calculated (**Table 2**). Sequenced clean reads were mapped against predicted transcripts of the *A. flavus* NRRL 3357 genome^1^ using Tophat v2.0.4 (Trapnell et al., 2009), and only unique matches were allowed. Transcript abundance (i.e., FPKM) were estimated using the HTSeq package. Differentially expressed genes were analyzed with the DESeq package (Anders and Huber, 2010), and both a twofold change cut-off and an adjusted *p*-value of ≤0.05 were established as thresholds. An enrichment analysis of differential expression was performed using the GOSeq R package (Young et al., 2010). GO terms (including cellular component, molecular function, and biological process) and Kyoto Encyclopedia of Genes and Genomes (KEGG) pathways were classified as significantly enriched among differentially expressed genes only when their Benjamini adjusted *p*-values were ≤0.05. All the RNA-seq data had been stored in GEO database with an ID of GSE107025.

**Table 2:** Summary of RNA-Seq data.

Sample	Raw reads	Clean reads	Clean bases	Error rate (%)	Q20 (%)	Q30 (%)	GC content (%)
WTYES	30430096	28722480	4.31G	0.02	95.62	89.42	52.3
WTYES	26018734	25164896	3.77G	0.02	95.41	88.94	52.18
WTYES	33291592	32246484	4.84G	0.01	97.45	93.38	52.38
DAfRafA	27344356	26437012	3.97G	0.02	95.36	88.9	52.15
DAfRafA	27222764	26268644	3.94G	0.02	95.39	88.94	52.3
DAfRafA	25355708	24273186	3.64G	0.03	95.04	88.26	52.36
DAfStuA	23060848	22148982	3.32G	0.02	95.32	88.83	52.75
DAfStuA	28387068	27318602	4.1G	0.01	97.47	93.41	52.53
DAfStuA	30533304	29493780	4.42G	0.01	97.41	93.33	52.75
WTYEP	23429420	21992086	3.3G	0.02	96.67	91.59	51.64
WTYEP	23791918	22660542	3.4G	0.02	97.2	92.76	52.18
WTYEP	23052088	21889226	3.28G	0.02	97.11	92.62	52.32

### Real Time Quantitative PCR

cDNA was synthesized from above mRNA sample by using the RevertAid RT Reverse Transcription Kit (Thermo Scientific, United States) following the manufacture’s instruction. Real time quantitative PCR (qPCR) was performed on a PikoReal Real-Time PCR System (Thermo Scientific, Inc.). All utilized primers were listed in **Table 1**. The relative expression level of each gene was calculated using the 2^-ΔΔC_t_^ method and the expression level of actin encoding gene was used as the internal control.

### Gene Co-expression Network

Co-expression network were constructed using a WGCNA R package (Langfelder and Horvath, 2008). The weighted matrix of pair-wise connection strengths (module) was built and genes were grouped into modules by hierarchical clustering. The power β was used to calculate the correlation coefficients and β = 14 was set as the saturation level for a soft threshold. These networks were then visualized using Cytoscape software.^2^

### Statistical Analysis

The significance of the data was tested using the Student’s *t*-test. A *p*-value of ≤0.05 was considered as significantly different.

## Results

### Comparison of Transcriptome of *A. flavus* Between AF Inducing and Non-inducing Conditions

**FIGURE 1:**
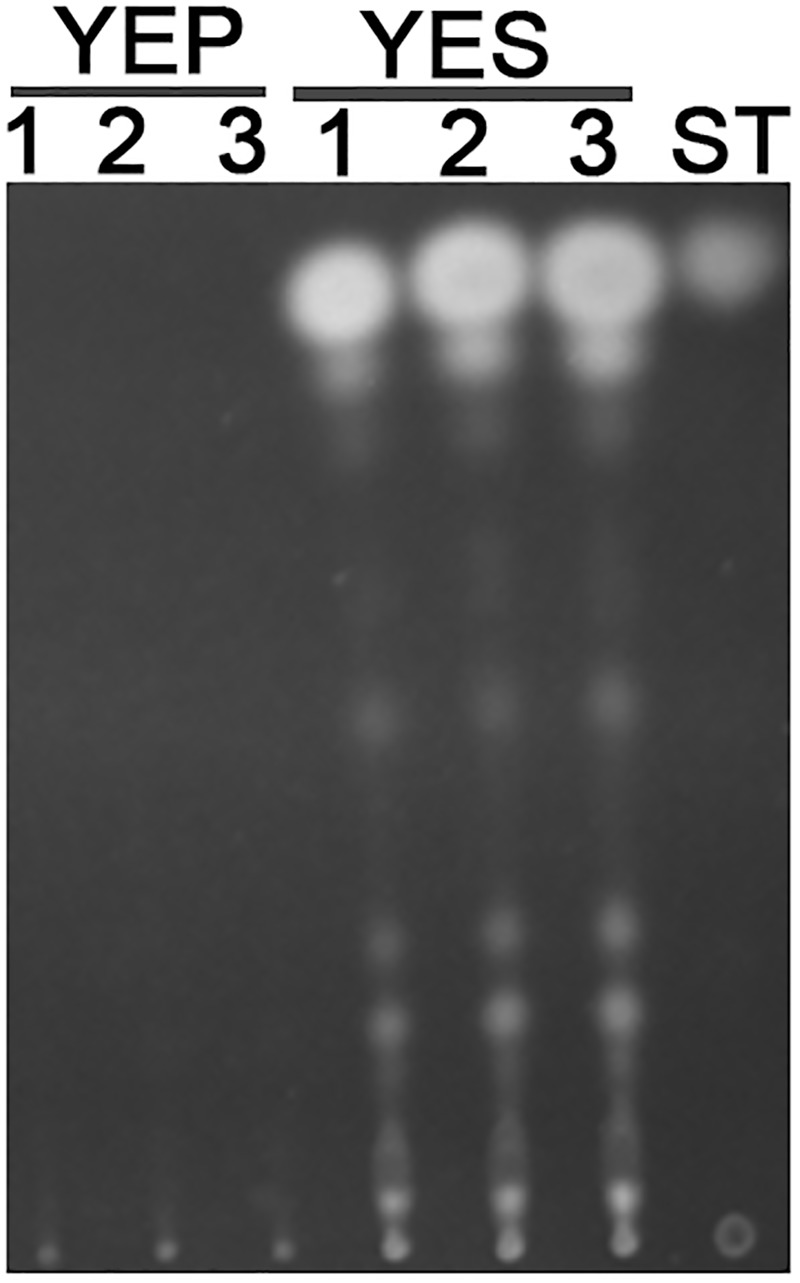
Aflatoxin production in YEP and YES. Conidia of *Aspergillus flavus* WT strain were grown in YEP and YES liquid for 7 days, and then 1.5 mL cultures were extracted by chloroform. TLC analysis of extracts from both YES and YEP, along with an AFB1 standard.

**FIGURE 2:**
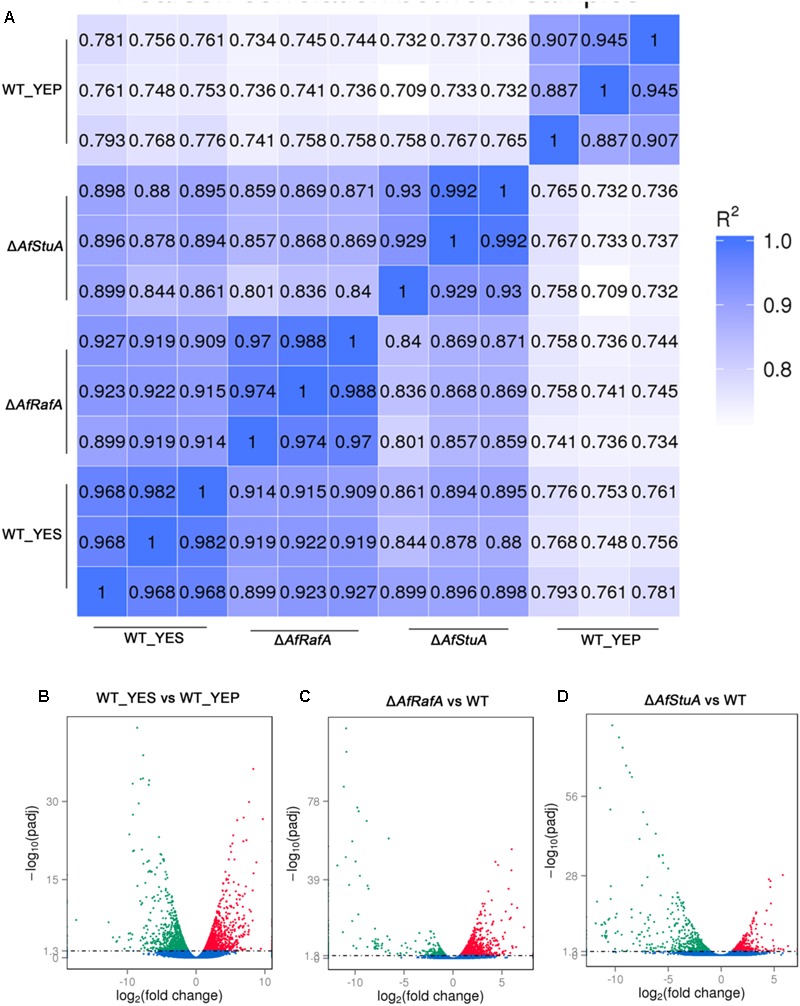
Analysis of differentially expressed genes in different conditions and various strains. **(A)** The Pearson correlation index among three biological repeats was calculated using R software. **(B)** Number of genes showed upregulated and downregulated expression of WT strain cultured in YEP vs. in YES; **(C)** Number of genes showed upregulated and downregulated expression in Δ*AfRafA* vs. WT; **(D)** Number of genes showed upregulated and downregulated expression in Δ*AfStuA* vs. WT.

Previous studies reported that YES strongly stimulate the biosynthesis of AF but YEP could not (Fountain et al., 2015). Since then, YES and YEP were defined as the AF conducing and non-conducing condition, respectively. In this study, we cultured the *A. flavus* WT strains in YES and YEP for 7 days, and the AF production was determined. As shown in **Figure 1**, YES induce a significant production of AF (11.5 ± 0.5 mg/10 mL cultures). However, no AF could be detectable in YEP, which was consistent with the previous report (Fountain et al., 2015), and confirmed that YES and YEP were AF conducing and non-conducing conditions, respectively. To understand the molecular mechanism underlying that YES but not YEP stimulates the AF biosynthesis, the transcriptome profiles of *A. flavus* cultured in both YES and YEP were comprehensively explored. The 48 h RNA was extracted and analyzed via high-throughput sequencing. High reproducible RNA-seq data were obtained from three biological replicates per culturing condition with Pearson correlation coefficients above 0.96 and 0.88 for YES and YEP, respectively (**Figure 2A**). In total, we obtained >3.3G clean bases with error rate ≤0.02 for each biological repeat of both two samples via deep sequencing. Quantifying the expected number of fragments per kilobase of transcript sequence per millions base pairs sequenced (FPKM), it was found that 2374 genes were differential expressed (DGE) with fold changes ≥2 as a threshold by comparing the transcriptomes between in YES and YEP. Among them, expression of 1336 genes were up-regulated, and 1038 genes were down-regulated in YES relative to in YEP (**Figure 2B** and **Supplementary Table S1**). Considering that seventeen percentage of *A. flavus* genome showed differential expression in YES vs. YEP, implying that these two media activate significantly different transcriptomes.

### Genome-Wide Identification of Gene Functions and Metabolic Pathways That Are Responsive to YES Induction, Regulation by AfRafA and AfStuA

**FIGURE 3:**
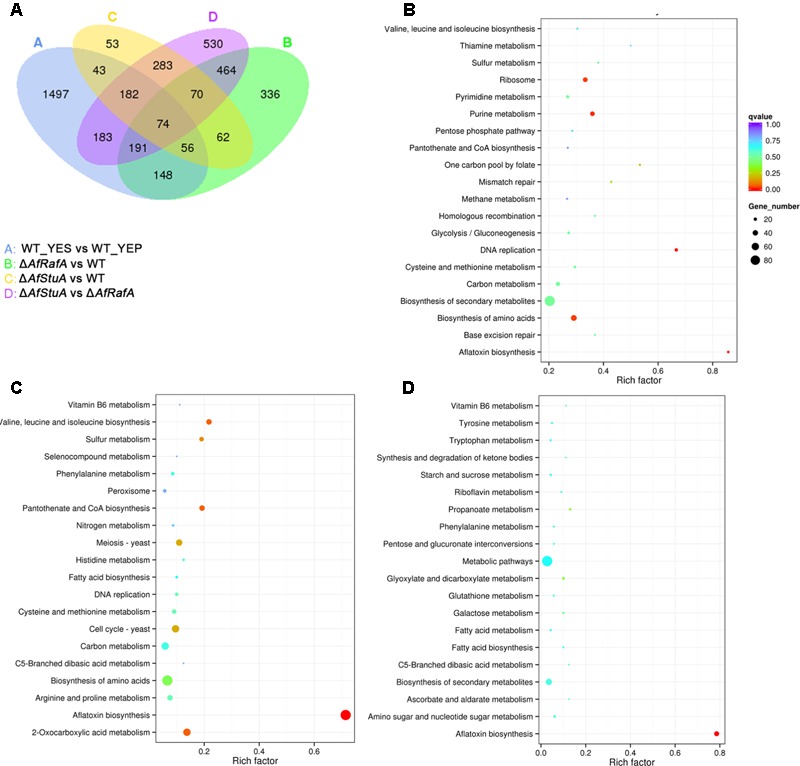
Kyoto Encyclopedia of Genes and Genomes (KEGG) enrichment analysis of differential expression genes. **(A)** Venn diagram of differential expression genes. **(B)** Enrichment analysis of genes upregulated above twofold in WT strain cultured in YES vs. in YEP; **(C)** Enrichment analysis of genes upregulated above twofold in Δ*AfRafA* vs. WT; **(D)** Enrichment analysis of genes upregulated above twofold in Δ*AfStuA* vs. WT.

Recently, we identified two novel APSES transcription factors (TFs), AfRafA and AfStuA, as important and essential activators for AF biosynthesis, respectively (Yao et al., 2017). To comprehensively understand their function in *A. flavus*, it is necessary to systematically identify both gene function and cellular processes they target. RNA-seq was performed to illustrate the gene landscapes regulated by AfRafA and AfStuA. The 48 h RNA samples of Δ*AfRafA* and Δ*AfStuA* under the same culture conditions as WT in YES were extracted and analyzed via sequencing. High reproducible RNA-seq data were obtained from three biological replicates per strain with Pearson correlation coefficients above 0.97 and 0.92 for Δ*AfRafA* and Δ*AfStuA*, respectively (**Figure 2A**). In total, we obtained >3.5G clean bases with error rate ≤0.02 and Q20 > 95% for each biological repeat of any strain via deep sequencing. Furthermore, by quantifying the FPKM, we found that 1401 genes were differential expressed when the transcriptomes were compared between Δ*AfRafA* and WT (**Supplementary Table S2**). These include 996 upregulated and 405 downregulated genes (**Figure 2C**). At the same time, a total of only 823 differentially expressed genes (DGE) were identified in Δ*AfStuA* vs. WT with identical threshold described above, including 356 upregulated and 467 downregulated genes (**Figure 2D** and **Supplementary Table S3**), suggesting that compared to AfStuA, AfRafA might play a more wide-range regulatory role in *A. flavus*. The Venn diagram showed that AfRafA and AfStuA shared 262 differential expression genes (**Figure 3A**), suggesting that these two proteins play overlapping roles in cellular regulation, which is further supported by their similar roles in AF and pathogenesis described previously (Yao et al., 2017). However, 1139 genes were subjected to AfRafA-specific regulation, while AfStuA uniquely controlled 561 genes (**Figure 3A**). It was also found that 1497 DGEs only showed increased or decreased expression in YES vs. YEP, unaffected by AfRaf or AfStuA. Functional enrichment of KEGG pathway of the DGEs between the deletion and WT strains, as well as AF-conducing vs. non-conducing conditions were performed, and the results were displayed in **Figures 3B–D**. Enriched analyses of the upregulated DGEs in YES uncovered that AF biosynthesis (afv00254) was significantly enriched, which accorded with our expectation (**Figure 3B**). Furthermore, DNA replication, purine metabolism, ribosome, and biosynthesis of amino acids were also significantly activated in YES (**Figure 3B**), which implying that they might have roles in AF synthesis. In addition, the downregulated DGEs were significantly enriched in valine, leucine and isoleucine degradation, penicillin and cephalosporin biosynthesis, and alpha-linolenic acid metabolism. As expected, the most downregulated genes in both Δ*AfRafA* vs. WT and Δ*AfStuA* vs. WT were also responsible for the pathway of AF biosynthesis (**Figures 3C,D**), further confirming that these two TFs function as key regulators for activating this toxin biosynthesis. These results are in well agreement with our previous AF production assays (Yao et al., 2017). Likewise, a number of metabolic processes, including valine, leucine and isoleucine biosynthesis, 2-oxocarboxylic acid metabolism, and pantothenate and CoA biosynthesis, which might relate to AF and other SMs biosynthesis were downregulated in Δ*AfRafA* vs. WT (**Figure 3C**). Correspondingly, the degradation of valine, leucine, and isoleucine was significantly upregulated in Δ*AfRafA* vs. WT. In addition, non-homologous end-joining and homologous recombination were markedly activated in Δ*AfRafA*, suggesting that AfRafA possibly involve in the DNA repair process. More interestingly, only AF biosynthesis was enriched in downregulated DGEs in Δ*AfStuA* vs. WT (**Figure 3D**), confirming that AfStuA functioned as essential AF-specific regulator. On the contrary, biosynthesis of other secondary metabolites showed upregulation in Δ*AfStuA* vs. WT, indicating a block of AF synthesis in Δ*AfStuA* would promote the redirection of metabolic flux to synthesize other SMs.

### Co-expression Network Analysis

**FIGURE 4:**
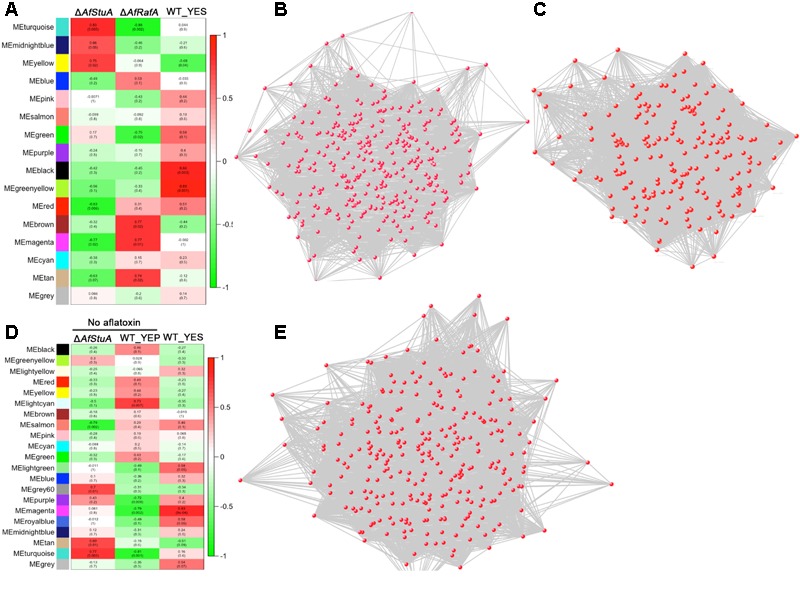
Weighted co-expression gene network. **(A)** Relationship between phenotype and consensus module eigengenes obtained by WGCNA for differentially expressed genes in Δ*AfRafA* vs. WT and Δ*AfStuA* vs. WT. **(B)** Gene network of MEblack module. **(C)** Gene network of MEgreenyellow module. **(D)** Relationship between phenotype and consensus module eigengenes obtained by WGCNA for differentially expressed genes in YES vs. YEP and Δ*AfStuA* vs. WT. **(E)** Gene network of MEmagenta module.

To explore the relationship between AfRafA and AfStuA regulons, a network analysis was determined by WGCNA (Langfelder and Horvath, 2008). According to the level of AFB1 production, we set phenotype of WT, Δ*AfRafA*, and Δ*AfStuA* as 1, 0.5, and 0, respectively. A gene set with differential expression were clustered into several modules labeling as distinct colors. Two modules (MEblack and MEgreenyellow) showed the highest correction with phenotype (*R*^2^ = 0.86 and *R*^2^ = 0.89). Three hundred and twelve genes of MEblack module were interacted to form the network **Figure 4B**. GO enrichment analysis of these genes showed that ribosome (GO:0005840), branched-chain amino acid biosynthetic process (GO:0009082) and translation (GO:0006412) were identified. Network from genes from MEgreenyellow module was shown in **Figure 4C** and no GO term was significantly enriched. No AF could be detected in YEP-cultured WT and YES-cultured Δ*AfStuA*, it is interesting to uncover general mechanism for aflatoxigenic phenotype. Analysis of all RNA-seq data of WT_YES, WT_YEP, and Δ*AfStuA* were cluster into 21 modules. One module (colored as MEmagenta) had a significant correction with phenotype (**Figure 4D**). Two hundred and eighty-seven genes from this module generated a gene co-expression network (**Figure 4E**). Among these genes, branched-chain amino acid biosynthetic process (GO:0009082), mitochondrial pyruvate transport (GO:0006850), signal peptide processing (GO:0006465) were enriched. It was worth to note that mitochondrial pyruvate transport and signal peptide processing potentially implicated in AF biosynthesis was proposed for the first time.

### Most Up-regulated Genes in YES vs. YEP Involve in Carbon and Nitrogen Metabolism

Analyses of the top twenty in upregulated DGEs in YES vs. YEP showed that most of them have roles in the nitrogen and carbon metabolism. Two ammonium transporters genes (*AFLA_108260* and *AFLA_130040*) were increased by above fivefold and sixfold under AF conducing condition compared with growth in non-conducing one. Likewise, the glutamate synthase gene (AFLA_022340), glutamate/phenylalanine/leucine/valine dehydrogenase gene (AFLA_113320), the amino acid transporter gene (*AFLA_073030*) were also up-regulated above fivefold in the AF conducing condition. Three most upregulated genes (7- ∼8-fold) were involved in carbon metabolism including alcohol dehydrogenase (*AFLA_097820*), carbohydrate kinase (*AFLA_097830*), glyceraldehyde 3-phosphate dehydrogenase (*AFLA_042390*). In addition, lipopolysaccharide-modifying protein (*AFLA_002000*), centromere protein V (*AFLA_096180*), isoprenoid synthase (*AFLA_042370*), O-methyltransferase (*AFLA_016120*), a TF with winged helix-turn-helix DNA-binding domain (*AFLA_016130*), antibiotic biosynthesis monooxygenase (*AFLA_121090*), phosphoesterase (*AFLA_050610*), and five genes with unknown function were identified, their roles in AF biosynthesis required further characterization. To summarize briefly, gene expression profiles of *A. flavus* strain cultures from YES and YEP were compared, and confirmed that AF biosynthesis, and genes related with carbon and nitrogen metabolism were significantly up-regulated in YES vs. YEP.

### Monooxygenase Subject to Specific Regulation of AfStuA

Intriguingly, the monooxygenase activity (GO:0004497) was significantly enriched when comparing Δ*AfStuA* and WT transcriptomes. In fact, 33 of 116 genes that encode cytochrome P450 monooxygenase showed differential expression (**Supplementary Table S4**). Among these, 25 genes were down-regulated, and four genes were up-regulated in Δ*AfStuA*. Remarkably, nine monooxygenase genes (*aflX, aflW, aflV, aflQ, aflI, aflL, aflG, aflN*, and *aflCa*) from the AF cluster were concurrently and strongly downregulated in Δ*AfStuA*. These results suggest a possibility that a variety of monooxygenases targeted by AfStuA, might contribute to the defective phenotype of Δ*AfStuA*. Consistent with this result is a recent study, which reported cytochrome P450 monooxygenases to be widely involved in various cellular processes, including fungal development, secondary metabolism, and virulence in the plant pathogen *Fusarium graminearum* (Shin et al., 2017). In summary, these data demonstrated that AfStuA exerted extensive regulatory roles in cellular primary and secondary metabolism, possibly by modulating the expression of various monooxygenases.

### Co-regulated Genes by AfRafA and AfStuA Are Mainly Enzymes Functioning at Early and Middle Stages in AF Biosynthesis

**FIGURE 5:**
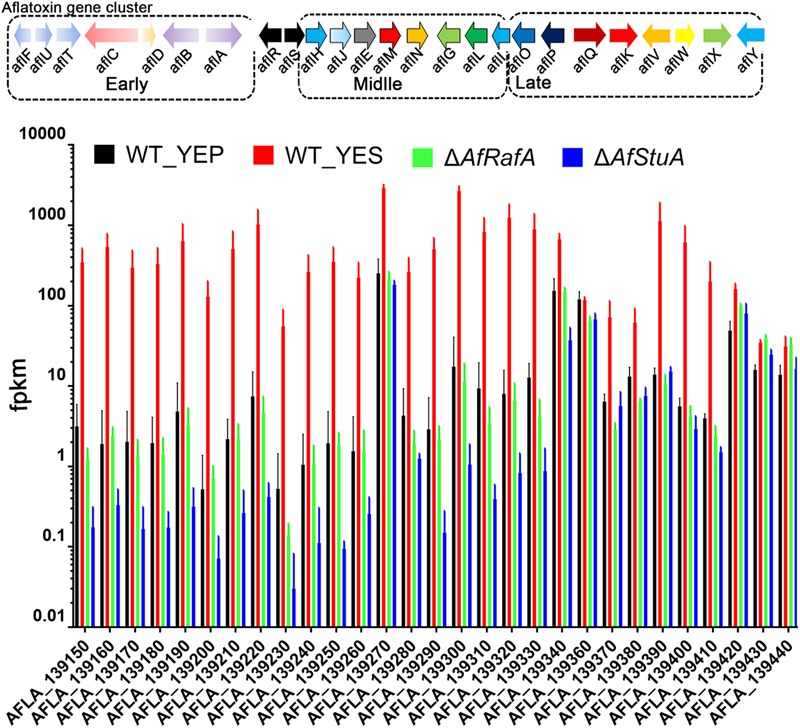
Differential roles for AfRafA and AfStuA in the regulation expression of different stage aflatoxin genes. Expression levels (fpkm) for all 29 aflatoxin genes in Δ*AfRafA* and in Δ*AfStuA*, as well as WT under YES and YEP were compared and grouped into three stages according to the roles of their protein product in making aflatoxin.

To fully understand the role of AfRafA and AfStuA in the activation of the AF gene cluster, the expression of 29 genes that were responsible for the generation of AF were selected out and compared as illustrated in **Figure 4**. A previous study reported that the enzymatic reactions, which are responsible for AF synthesis could be divided into three stages: early, middle, and late stage (**Figure 5**). AflD, AflM, and AflP in *A. flavus*, as well as NorA, Ver-1, and OmtA in *A. nidulans* were representatives in the three stages, respectively (Chanda et al., 2009). Interestingly, genes encoding early enzymes showed a more severe downregulation of their transcription in both deletion strains relative to those decoding enzymes that functioning at middle and late stages, which was reflected by the almost undetectable mRNA of most of these early genes in Δ*AfStuA*. As expected, almost all AF genes (not including *AflR*) were significantly induced in YES in comparison to in YEP (**Figure 5**). And, most of the AF cluster genes in this group conformed an expression pattern that was highest in WT, lower in Δ*AfRafA*, and even lower or completely block in Δ*AfStuA*, indicating that AfStuA played a more significant regulatory role in AF biosynthesis relative to AfRafA. However, the three late enzymes encoding genes *aflW, aflX*, and *aflY* showed a WT-level expression in Δ*AfRafA* and Δ*AfStuA*. In summary, AfStuA and AfRafA played key roles in activating the AF gene cluster at the initial phase, also demonstrating that expression of the early-stage enzyme genes for AF synthesis were co-regulated by these two TFs.

### Expression of Multiple AF Regulators Are Responsive to YES, or AfRafA and AfStuA Regulation

**FIGURE 6:**
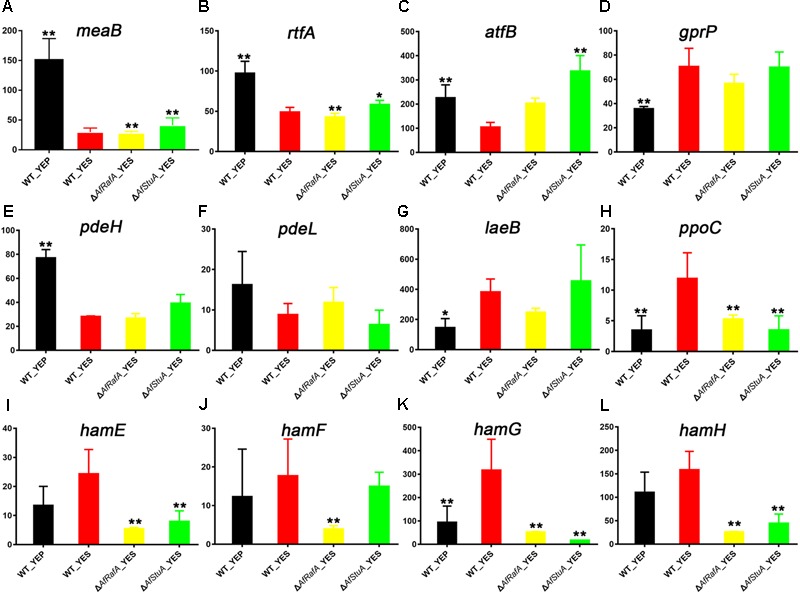
Expression of known aflatoxin regulators in various strains. Expression level (fpkm) for 12 aflatoxin regulator genes in Δ*AfRafA* and in Δ*AfStuA*, as well as WT under YES and YEP were compared, including *meaB* **(A)**, *rtfA* **(B)**, *atfB* **(C)**, *gprP* **(D)**, *pdeH* **(E)**, *pdeL* **(F)**, *laeB* **(G)**, *ppoC* **(H)**, *hamE* **(L)**, *hamF* **(M)**, *hamG* **(N)**, and *hamH* **(L)**. ^∗^*p* ≤ 0.05, ^∗∗^*p* ≤ 0.01.

Interestingly, 12 known AF regulators were identified by our comparative transcriptome analysis, because their expression pattern showed differential response to culturing condition, and AfRafA and AfStuA regulation. Two transcription regulators MeaB and RtfA, was previously identified as negative regulators (Amaike et al., 2013; Lohmar et al., 2016), and their expression levels were significantly downregulated in YES when compared with in YEP, but seems not to be regulated by AfRafA or AfStuA in this study (**Figures 6A,B**). Unexpectedly, the bZIP TF AtfB, positively regulating AF biosynthesis (Wee et al., 2017), its transcription level was downregulated by twofold in YES vs. in YEP, whereas upregulated by threefold in Δ*AfStuA* mutant in compassion to WT (**Figure 6C**), implying that AfStuA regulates AF in an AtfB-independent way. G protein-coupled receptor gene *gprP* was up-regulated in all strains used in YES compared with WT cultured in YEP (**Figure 6D**), and its encoding protein negatively control the AF biosynthesis (Affeldt et al., 2014). On the contrary, expression of two phosphodiesterase genes *pdeH* and *pedL* were downregulated significantly in YES in each mutant strain relative to YEP (**Figures 6E,F**), which was consistent with their negative roles in toxin formation (Yang et al., 2017). More recently, the novel protein LaeB was demonstrated to be crucially required for both sterigmatocystin and AFs biosynthesis in *A. nidulans* and *A. flavus*, respectively (Pfannenstiel et al., 2017). Unexpectedly, it was showed that expression of laeB was markedly upregulated in YES, and upregulated by AfStuA without statistical significance, but not by AfRafA (**Figure 6G**). Oxylipin-generating dioxygenases include PpoC, were reported to involve in the AF synthesis (Brown et al., 2009). In accordance with the expression pattern of AF cluster, expression of *ppoC* was the highest in WT under YES media, lower in YEP or Δ*AfRafA*, and further lower in Δ*AfStuA* (**Figure 6H**). Recently, it was reported that genes responsible for hyphal anastomosis regulated the biosynthesis of AF in the LaeA-dependent manner. Especially, deletion of *hamF, hamG, hamH*, or *hamI* resulted in an almost abolish of AF biosynthesis, similar to the phenotype of Δ*laeA* (Zhao et al., 2017). Interestingly, our data suggested a role of these proteins in the induction of AF in an AfRafA or AfStuA, or both dependent way. As for *hamE*, YES have negligible impact on its expression, but its expression level was greatly decreased in Δ*AfRafA* or Δ*AfStuA* (**Figure 6I**). Expression of *hamF* was not affected by YES induction or AfStuA activation, showing a specific downregulation in Δ*AfRafA* (**Figure 6J**). *hamG* was expressed at the highest level in WT under YES, decreased threefold in WT under YEP, and further downregulated in Δ*AfRafA* (sixfold) and Δ*AfStuA* (above 10-fold) (**Figure 6K**). Expression of *hamH* was responsive to the regulation of AfRafA and AfStuA, regardless of media used (**Figure 6L**). All together, these observations indicated that AfRafA and AfStuA played a divergent role in the control expression of hyphal anastomosis genes, and their mediated AF biosynthesis. Totally, 12 important AF regulators were identified by the comparative transcriptome analysis, indicating that our comparative transcriptomic strategy is efficient to distinguish the potential targets with roles in AF synthesis.

### A Novel LaeA-Like Methyltransferase Involves in the Control of AF Biosynthesis

**FIGURE 7:**
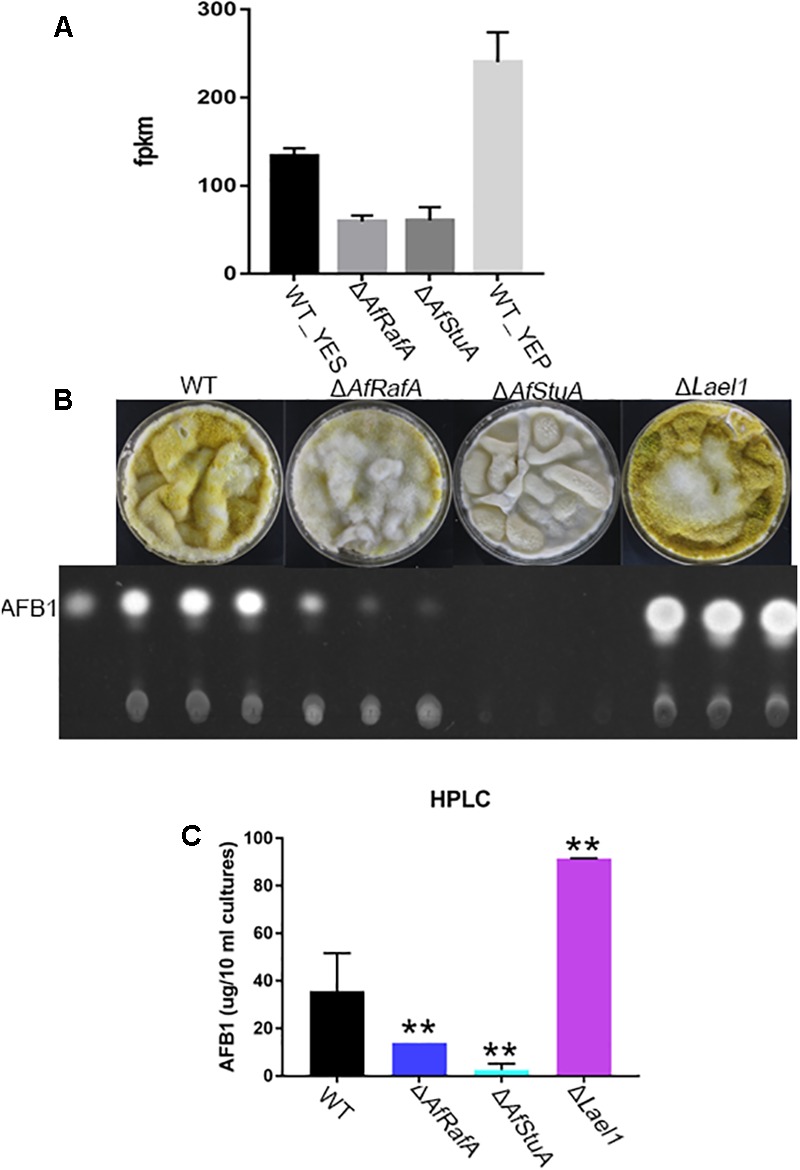
A novel LaeA-like methyltransferase Lael1 controls aflatoxin biosynthesis. **(A)** Expression level of methyltransferase gene *lael1* when cultured in YES and YEP and in Δ*AfRafA* and Δ*AfStuA*; **(B)** TLC analysis of aflatoxin of Δ*lael1*, Δ*AfRafA*, Δ*AfStuA*, and WT strains induced by YES; **(C)** HPLC analysis of aflatoxin titers of Δ*lael1*, Δ*AfRafA*, Δ*AfStuA*, and WT strains, ^∗∗^*p* ≤ 0.01.

Beyond known AF regulators, a number of novel candidate genes that might involve in AF were also identified in our transcriptomic analysis (see Discussion). Intriguingly, it was suggested that expression of one LaeA-like methyltransferases genes *AFLA_121330* (thereafter named as *lael1*) was significantly downregulated in YES relative to in YEP, and further subjected to regulation of both AfRafA and AfStuA (**Figure 7A**). In addition, regulated expression pattern of *lael1* by media or regulators of AfRafA and AfStuA was further confirmed by qPCR analyses (**Figure 7A**), implying that it might have roles in the induction of biosynthesis of AFs. To examine the role of Lael1 in AF, we generated the gene knockout mutant strain for gene *lael1*. After two rounds of genetic transformation, three deletion transformants were obtained and verified (**Supplementary Figure S1**). And then, the phenotype and the AF production of these mutants were determined. Deletion of *lael1* did not affect colony extension or conidiation when growth either on PDA or GMM agar plate supplemented with glutamine (data not shown), suggesting that Lael1 did not involve in the regulation of fungal growth and development. However, the AF titers of Δ*lael1* were significantly increased in our assays (**Figures 7B,C**). Especially, Δ*lael1* produced threefold more AFB1 than that of WT when growth in YES liquid media. To confirm the effect of Lael1 on AF biosynthesis, the complementation strain (Lael1com) was constructed by re-introduction the expression cassette to Δ*lael1*. As expected, the complementation strain restored the AF production to wild-type level (data not shown). As expected, only trace AF could be detected in Δ*AfStuA*, and significantly reduced AF synthesis was detected in Δ*AfRafA* (**Figure 7C**). In addition, conidia of Δ*lael1* was more pigmented than those of other mutant and WT strains. Combined above, it was confirmed that Lael1 played crucial roles in suppression of the biosynthesis of AF, and its expression were activated by YEP but not YES, and further positively regulated by both AfRafA and AfStuA.

### Both AfRafA and AfStuA Function as Global Regulators and Affect the Expression of Multiple SMs Clusters

**FIGURE 8:**
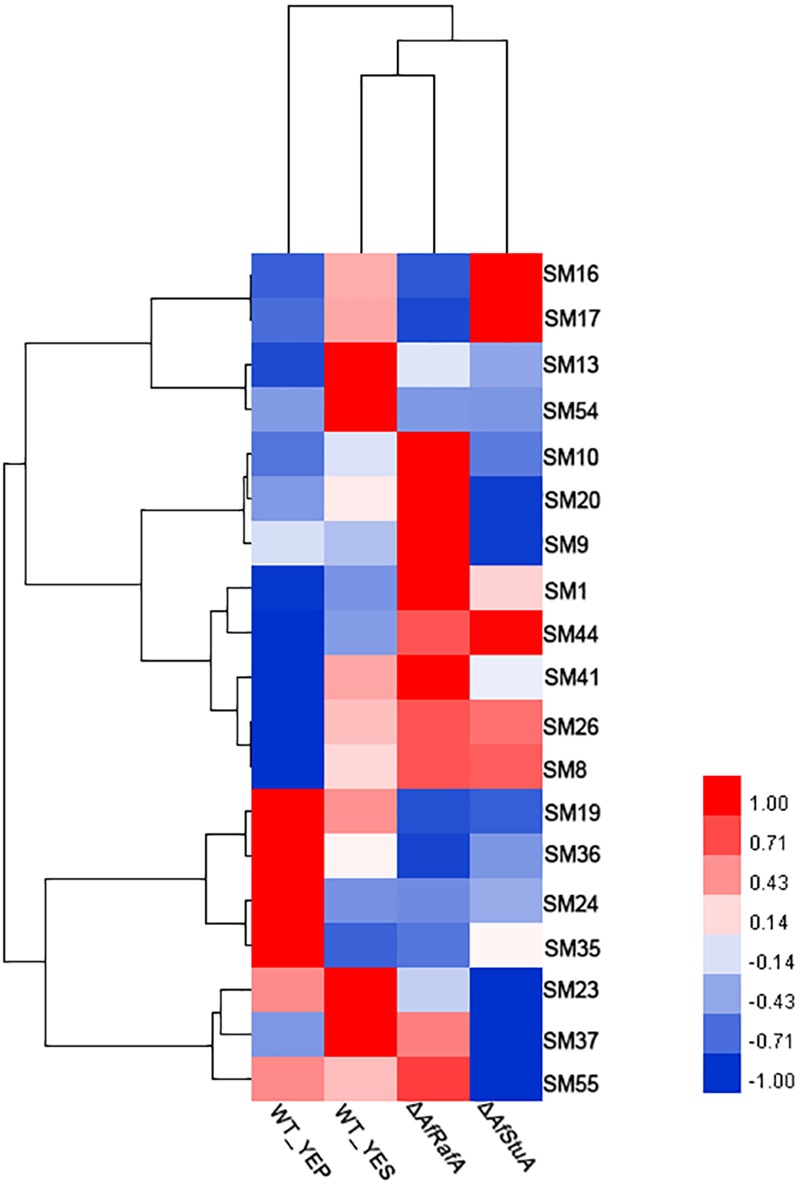
Heatmap of expression levels of secondary metabolism core genes in various strains. Expression level (fpkm) for 19 genes encoding the core enzymes for secondary metabolisms in Δ*AfRafA* and in Δ*AfStuA*, as well as WT under YES and YEP were clustered into three patterns and showed in the heatmap.

In addition to AF, the *A. flavus* genome harbors additional 54 SM clusters, however, most of their chemical nature remains unclear. To clarify the roles of AfRafA and AfStuA in other SMs, the expression pattern of SM core enzyme encoding genes were examined between in YEP and in YES, as well as in gene knockout mutants and in WT, and the results are hierarchically clustered as showed in **Figure 8**. In total, 19 gene clusters, each of which was responsible for the synthesis of at least one class of SMs, was expressed in at least one sample. All the SM core genes were clustered into three groups based on their mRNA abundance in Δ*AfRafA*, Δ*AfStuA*, and WT. One group containing four SM gene clusters displayed the highest expression level in WT, lower expression in Δ*AfRafA*, and much lower or even non-existent expression in Δ*AfStuA*. Among these, only SM54 had been identified and was an AF gene cluster. The core enzymes of SM13, SM16, and SM17 were non-ribosomal peptide synthetase (NRPS), L-ornithine-N5-oxygenase (SidA), and polyketide synthase (PKS)-like, respectively, implying that they might produce non-ribosomal peptides, siderophore, and unknown chemicals, respectively. Interestingly, the second group of SM genes shared an expression pattern that showed higher expression in Δ*AfRafA*, but lower or even a complete lack of expression in Δ*AfStuA* when compared to WT. This group includes SM9, SM10, SM20, SM37, and SM55. Among these, the product of SM55 has recently been characterized as cyclopiazonic acids, which was agreed with our previous results that AfRafA negatively and AfStuA positively regulated the biosynthesis of cyclopiazonic acid (Yao et al., 2017). The enzymes for synthesizing the backbone of SM9 and SM37, SM10, and SM20 were the NRPS, scytalone dehydratase, and PKS, respectively. Four SM clusters were specifically induced in YES relative to in YEP, including SM19, SM24, SM35, and SM36, harboring the core enzymes encoding dimethylallyl tryptophan synthase, NRPS, NRPS-like enzyme, and PKS-like enzyme, respectively. Markedly, most of the SMs showed lowest or even non-existent expression in Δ*AfStuA* vs. WT, suggesting that *AfStuA* likely function as a global activator of secondary metabolism. On the contrary, AfRafA played a negative role in the biosynthesis of many SMs, except for AF gene cluster. The knowledge obtained in this study would pave a novel way for activating the internally silent SM by over-expressing AfStuA or deleting AfRafA in our future natural product discovery in *A. flavus*.

## Discussion

Nitrogen metabolism links with AF biosynthesis (Reddy et al., 1979; Payne and Hagler, 1983; Bhatnagar et al., 1986; Ehrlich and Cotty, 2002; Wilkinson et al., 2007; Georgianna and Payne, 2009; Tudzynski, 2014). Payne and Hagler (1983) reported that casein strongly repress AF in both *A. flavus* and *Aspergillus parasiticus*. By analogy, in this study casein-derived peptone has a similar effect. Both casein and peptone enriched in amino acids were preferentially utilized as favored nitrogen resource. Metabolism of these favored nitrogen triggers *n*itrogen *m*etabolite *r*epression (NMR) results in expression of genes for metabolizing non-favored nitrogen are downregulated (Tudzynski, 2014). An increasing evidence demonstrated a regulatory role of NMR in the biosynthesis of secondary metabolism, including AF in filamentous fungi. Two TFs AreA and NmrA are central regulators in NMR. Chang et al. (2000) reported that AreA binds with the intergenic region between *aflR* and *aflS* to regulate *aflS* expression. Recently, NmrA was shown to strongly affect AF synthesis on glutamine and alanine media (Han et al., 2016). Relative to YEP, YES is poor in amino acids. Therefore, it was proposed that YEP mediates the nitrogen repression effect, but YES resulted in depression of AF gene cluster. In fact, YES induces a similar transcriptional response as shortage of amino acid, reflected by organonitrogen compound biosynthetic process (GO:1901566, Corrected *p*-value = 1.29E-11) and cellular amino acid biosynthetic process (GO:0008652, Corrected *p*-value = 9.72E-07) were significantly upregulated. Accordingly, metabolism of valine, leucine and isoleucine (*p*-value = 6.03E-05) and phenylalanine (*p*-value = 0.014072269) were downregulated. Further analysis indicated that three genes specially involved in proline biosynthesis (*AFLA_014050, AFLA_047450*, and *AFLA_047280*) were upregulated in YES. Aspartate has been shown to stimulate more AF biosynthesis than other nitrogen (Payne and Hagler, 1983). Bhatnagar et al. (1986) suggested that glutamine synthetase played an important regulatory role in biosynthesis of AFs by modulating glutamine level. Our recent results further supported that glutamine induces more AF production than other inorganic nitrogen and amino acids (Wang et al., 2017). Combined, it was proposed that glutamine is converted into acetate through alpha-ketoglutarate and then incorporated into AFs. Indeed, both glutamine synthetase gene *AFLA_022340* (converting glutamine into glutamate) and aminotransferase gene *AFLA_026470* (converting glutamate into 2-oxoglutarate) was upregulated 10- and 8-fold in YES vs. YEP, respectively. Interestingly, tryptophan metabolism mediated regulation of AF synthesis was different between in *A. flavus* and in *A. parasiticus* (Wilkinson et al., 2007). In addition, amino acid variety in crops could also influence severity of crop AF contamination (Senyuva et al., 2008). Altogether, nitrogen resource exerts a complex effect on AF biosynthesis, further characterization of nitrogen metabolism and regulatory genes in *A. flavus* are required.

Biosynthesis of AF is a highly regulated process; a hierarchy and interconnected network is involved. In the network, AflR function as a pathway-specific regulator, function at the basal level to activate AFB. Expression of *aflR* was downregulated in Δ*AfRafA*, or even completely inhibited in Δ*AfStuA* by our RNA-seq analysis, according well with our previous qPCR results (Yao et al., 2017), supporting that AfRafA and AfStuA function as a global regulator functioning upstream of AflR. In the middle, Lael1, as a methyltransferase, might activate *aflR* transcription by modification of chromatin structure, just as LaeA (Bok et al., 2006). In addition, our results support that expression of *Lael1* was subjected to regulation of AfStuA and AfRafA. Co-expression network analysis demonstrated that AfStuA and AfRafA control both the same and divergent cellular processes, suggesting a cross-talk between AfRafA regulon and AfStuA regulon.

One novel LaeA-like proteins Lael1 were demonstrated to have crucial roles in AF production, but appear not to affect other cellular processes, suggesting it might serve as an AF-specific regulator. Previously, as the SAM-dependent methyltransferase, LaeA positively regulated the biosynthesis of mycotoxin in all most filamentous fungi (Bok and Keller, 2004; Wu et al., 2012; Crespo-Sempere et al., 2013; Kim et al., 2013; Estiarte et al., 2016). However, our data supported a negative regulatory role of Lael1 in the biosynthesis of AFs. Likewise, LlmF, a LaeA-like methyltransferase, negatively control the sterigmatocystin synthesis by mediating the cellular location of VeA (Palmer et al., 2013). Genetic screen of *A. nidulans* LaeA-like methyltransferase proteins did not suggest a regulatory role for Lael1 ortholog in sterigmatocystin production (Palmer et al., 2013). It was widely accepted that biosynthesis of AF in *A. flavus* shares a same regulatory network to sterigmatocystin (the penultimate precursor of AF) in *A. nidulans*. However, our data highlighted a caution that knowledge obtained in sterigmatocystin biosynthesis in *A. nidulans* may not absolutely applied to biosynthesis of AF in *A. flavus*. Actually, this hypothesis was supported by several reports. Loss of the upstream development regulator FluG lead to a block of biosynthesis of sterigmatocystin in *A. nidulans* but not in *A. flavus* (Chang et al., 2012). Unlike in *A. nidulans*, the C_2_H_2_ TF RsrA do not impact the biosynthesis of secondary metabolism in *A. flavus* (Bok et al., 2014). In addition, extracellular pH causes a divergent effect on the biosynthesis of AF in *A. flavus* and *A. parasiticus*, and sterigmatocystin in *A. nidulans*, respectively (Ehrlich et al., 2005; Delgado-Virgen and Guzman-de-Pena, 2009).

The first step in the biosynthesis of AF is the formation of a hexanoyl starter unit from acetyl-CoA and malonyl-CoA (Minto and Townsend, 1997). In fact, pantothenate and CoA biosynthesis (afv00770) was more strongly expressed in AF-inducing condition than in non-inducing, on the contrary, significantly downregulated in the transcriptional profiles of Δ*AfRafA* and Δ*AfStuA*. It had been demonstrated that initiation of AF biosynthesis correlated with the activation of acetyl-CoA carboxylase by calmodulin or oxidative stress (Rao and Subramanyam, 2000; Narasaiah et al., 2006). Therefore, combined above results might inspired us that increasing biotechnologically the building block could be an efficient way to improve the production of polyketide-derived secondary metabolism.

By comparative transcriptomics, we identified a number of known and novel regulators required for AF biosynthesis. Comparing the composition, high concentration sucrose and MgSO_4_ in YES were replaced with peptone in YEP, implying sucrose and magnesium ion might facilitate AF synthesis. Indeed, metal ion, including magnesium showed an effect on the AF biosynthesis (Tiwari et al., 1986). To support the above hypothesis, sucrose had been shown to induce the AF synthesis in the concentration-dependent way (Llewellyn et al., 1980; Wilkinson et al., 2007). It would be more important to note that novel proteins with an identical expression pattern to AF cluster, potentially involve in the toxin biosynthesis. Eighteen candidate genes satisfy the criteria in our comparative transcriptome analysis, including two TFs – AFLA_084720 and NosA, five transporters – allantoate permease (AFLA_019420), ZIP Zinc transporter (AFLA_081920), pantothenate transporter (AFLA_108250), ammonium transporter (AFLA_108260), and efflux pump (AFLA_131810), two monooxygenase (AFLA_019430 and AFLA_138920), forkhead domain protein (AFLA_132980), NADH-cytochrome B5 reductase (AFLA_039650), pyridoxamine phosphate oxidase (AFLA_108810), fucose-specific lectin (AFLA_065960), neuroligin (AFLA_013540), and four genes with unknown function. Interestingly, Zhao et al. (2017) reported that NosA regulates *ham* genes and heterokaryon, virulence and AF production, while the function of these novel genes in *A. flavus* remain unidentified. Defining their possible roles in AF biosynthesis warrant further investigation. In conclusion, comparative transcriptome established here was an efficient strategy to identify the potential regulators for AF and other SMs biosynthesis.

## Author Contributions

GY and SW conceived and designed the work. GY, YF, YY, GM, ZF, and ZX performed the experiments, analyzed the data, and wrote the manuscript. SW revised the manuscript.

## Conflict of Interest Statement

The authors declare that the research was conducted in the absence of any commercial or financial relationships that could be construed as a potential conflict of interest.

## Footnotes

1 http://fungi.ensembl.org/Aspergillus_flavus/Info/Index

2 http://cytoscape.org/

## Supplementary Material

FIGURE S1 Verification of gene deletion of *Lael1*. **(A)** Scheme of gene deletion of *AFLA_121330*; **(B)** PCR verification of locus of knockout cassette; **(C)** RT-PCR verification gene loss of *AFLA_121330*.

TABLE S1 Differential expression genes in YES vs. YEP.

TABLE S2 Differential expression genes in Δ*AfRafA* vs. WT.

TABLE S3 Differential expression genes in Δ*AfStuA* vs. WT.

TABLE S4 Expression level of monooxygenase genes.
