# Supplementary figures and images for: Exploration of the Regulatory Mechanism of Secondary Metabolism by Comparative Transcriptomics in Aspergillus flavus
Source: Front Microbiol. 2018 Aug 7;9:1568. doi: 10.3389/fmicb.2018.01568 (PMC6090018; doi:10.3389/fmicb.2018.01568)

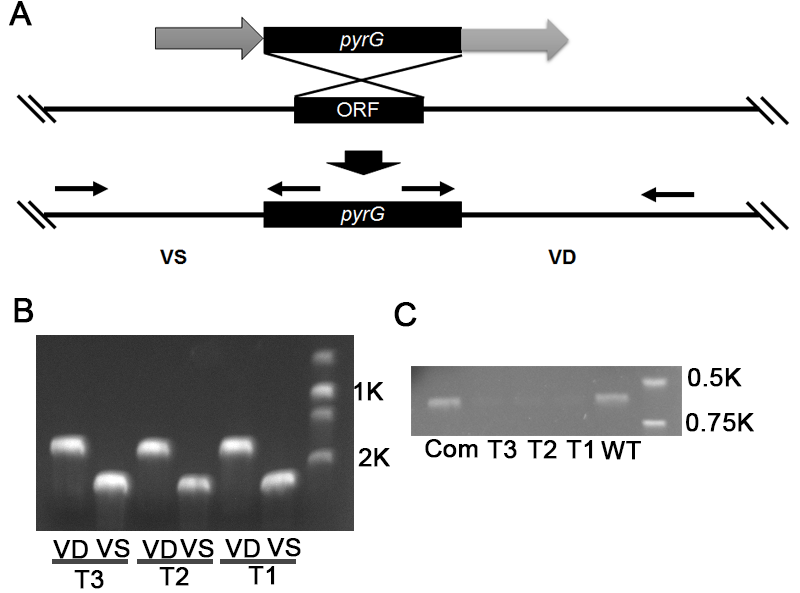

Supplement: FIGURE S1 — Verification of gene deletion of Lael1. (A) Scheme of gene deletion of AFLA_121330; (B) PCR verification of locus of knockout cassette; (C) RT-PCR verification gene loss of AFLA_121330. [file Image_1.TIF]
